# Jean-Luc Darlix: Renaissance scientist and retrovirologist *par excellence*

**DOI:** 10.1186/1742-4690-8-59

**Published:** 2011-07-18

**Authors:** Mark A Wainberg, Eric A Cohen

**Affiliations:** 1 McGill University AIDS Centre, Lady Davis Institute, Jewish General Hospital, Montreal, Quebec, Canada; 2 Institut de Recherches Cliniques, Université de Montréal, Montreal, Quebec, Canada

## Abstract

Jean-Luc Darlix was recently recognized with the first *Retrovirology *Lifetime Achievement Award on the occasion of his "retirement" symposium in Lyon.

## 

On June 1-2, 2011, a special symposium was organized at the Ecole Normale Superieure (ENS) in Lyon, France to mark the imminent retirement from that institution of its Head of Human Virology, Professor Jean-Luc Darlix. The symposium featured scientific presentations by both French and international scientists who came from countries throughout the world to pay tribute to their friend and colleague and to celebrate with him his legendary and fulfilling career in virology and biochemistry that has spanned more than four decades. During this time, Jean-Luc Darlix has been responsible for the training of more than 50 graduate students and post-doctoral fellows, many of whom are now outstanding scientists in their own right and who hold independent research positions both in France and elsewhere. The occasion was marked by a special recognition given to Jean-Luc by the Editors of *Retrovirology*. Notably, Professor Darlix was a founding member of the editorial board of *Retrovirology *and has served tirelessly on the board for the past eight years. He has also authored eleven *Retrovirology *papers including recent contributions [1,2]. To honor his multiple and original achievements over many years toward the advancement of retrovirus research, Jean-Luc received the first ever *Retrovirology *Lifetime Achievement Award (Figure 1).

**Figure 1:**
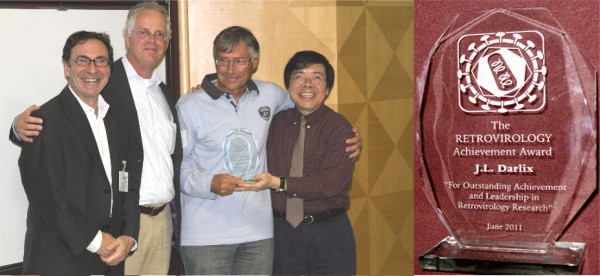
**Presentation of the Retrovirology Lifetime Achievement Award**. (left) From left: Mark A. Wainberg, Ben Berkhout (Editors), Jean-Luc Darlix (award recipient) and Kuan-Teh Jeang (Editor-in-Chief). (right) Photograph of the *Retrovirology *Lifetime Achievement Award trophy.

Jean-Luc obtained his PhD under the supervision of P Fromageot and F Gros while studying "In Vitro Transcription of DNA by Using Purine Nucleotide Analogs". Subsequently, he performed post-doctoral research in both the USA and in Switzerland, prior to taking up his first faculty position in 1977 at the University of Geneva where he studied both Rous Sarcoma virus(RSV) and Moloney Leukemia virus (MLV) as models of viral replication. It was during this period that Jean-Luc published some of the first findings anywhere on retroviral diversity and this work infused him with the passion that would define his career as someone who wanted to understand the details of retroviral replication in a manner that other scientists had never been able to accomplish. When HIV-1 was identified in 1983 as the causative agent of AIDS, Jean-Luc was well placed to take on the study of this latter virus.

In 1985, Jean-Luc moved back to France to take up an independent faculty position in Toulouse, where he broadened his work on RSV and MLV, before ultimately turning much of his focus to the study of HIV. In 1990, he accepted the position at ENS Lyon that he has occupied to this time, all the while moving up the ranks at the Ecole Normale Superieure through promotions and the assumption of major administrative responsibilities. Remarkably, Jean-Luc has maintained a track record of outstanding scientific achievement throughout his career with over 200 major publications in excellent peer-reviewed journals. His work is among the most highly cited of any retrovirologist in the world, and he is responsible for a multitude of original scientific observations. Chief among these is his seminal work on the role of the viral Nucleocapsid (NC) protein in the promotion of retroviral reverse transcription and as a chaperone protein that interacts with both viral nucleic acid as well as other viral proteins. As such, Jean-Luc was the first scientist to truly understand that NC played crucial roles in both of the pre- as well as post-integrational phases of the retroviral life cycle. This has given rise to efforts to develop drugs that might antagonize the NC protein, particularly that of HIV, and Jean-Luc, together with French and American colleagues, played a key role in determining that the two Zinc finger motifs of NC might represent the most likely avenue for anti-NC drug development. These efforts continue today in laboratories throughout the world.

It is, however, fair to point out that Jean-Luc's work has also directly contributed to the development of the nucleoside analog compounds that are today used to treat HIV infections. His fundamental research over many years on reverse transcription and viral diversity helped to establish the techniques necessary to screen antiretroviral drugs in both viral replication as well as in cell-free reverse transcriptase reactions. And, his understanding of retroviral diversification also enabled Jean-Luc to be one of the first to predict the limitations of antiretroviral therapy and the emergence of HIV drug resistance.

In addition to his role as a mentor to his own students and post-doctoral fellows, Jean-Luc has also been a wonderful colleague and friend to countless numbers of scientists. His lab was always open to junior scientists who were sent to Lyon to learn techniques for assessing retroviral replication from the members of Jean-Luc's team. His published bibliography shows a multitude of papers that he co-authored with other scientists in the field. This provides further testimony to his ability to befriend others as well as to work in harmony through the sharing of laboratory constructs and reagents. In addition, Jean-Luc served over many years as a member of grants panels in France through the Agence Nationale pour la Recherche contre le SIDA (ANRS) and Sidaction, as well as in the United States and Canada. His colleagues at grants panel meetings will recollect that Jean-Luc always went to extraordinary lengths to ensure that his clothes were the most muti-colored and loud of anyone on the committee, a dress code that sometimes also carried over to the laboratory.

To this day, Jean-Luc remains passionate about scientific discovery and feels a sense of excitement each time one of his papers is published. Nothing gives him more pleasure than discussing and even arguing about the intricacies of retroviral replication with colleagues and friends. But, Jean-Luc is also an exceptionally well-rounded individual with other interests as well. Of course, he remains devoted to his family and four children and takes great pride in their accomplishments. In addition, Jean-Luc is a noted gourmet who not only enjoys and can provide excellent advice on fine cuisine, but he is himself an experienced chef, who time permitting, can turn out as fine a meal as any virologist in the world. And, Jean-Luc is never afraid to turn back a bottle of wine in a restaurant, if his uncanny sense of smell and taste tells him that a bottle has "turned".

But, of course, no tribute to Jean-Luc could be complete without mentioning his accomplishments as a mountaineer and climber who has scaled peaks in the Alps, Rockies, Andes, and elsewhere, while of course appropriately dressed to withstand cold and snow conditions (Figure 2). Indeed, Jean-Luc holds advanced degrees in mountaineering from schools in France, and is also a teacher of mountaineering arts. Everyone who knows Jean-Luc is familiar with the photographs that have been taken of him at the tops of snow-capped mountain peaks. It is remarkable how he found the time to indulge his passion for climbing while maintaining devotion to his laboratory as well as an outstanding publication record.

**Figure 2:**
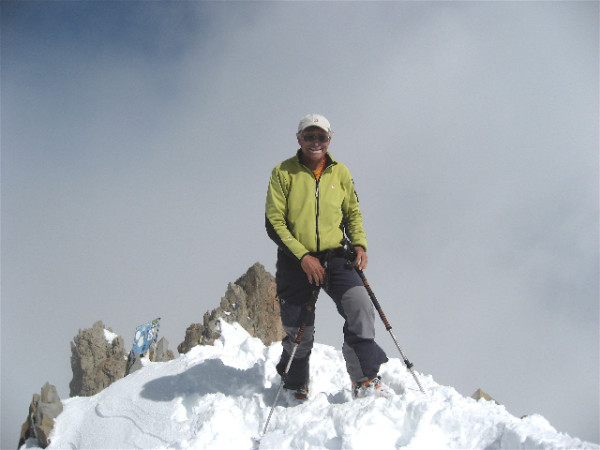
**Photograph of Professor Jean-Luc Darlix in mountain gear**.

No one who knows Jean-Luc believes that his scientific career will slow down anytime soon. Rather, it is simply entering a new phase of collaborations and potential industrial consulting that will continue to yield important accomplishments and research publications for many years to come. A few of Jean-Luc's favourite papers are listed in the reference list below [3-10].
